# Neurons Specifically Activated by Fear Learning in Lateral Amygdala Display Increased Synaptic Strength

**DOI:** 10.1523/ENEURO.0114-18.2018

**Published:** 2018-07-04

**Authors:** C. W. Butler, Y. M. Wilson, J. Oyrer, T. J. Karle, S. Petrou, J. M. Gunnersen, M. Murphy, C. A. Reid

**Affiliations:** 1 Department of Anatomy and Neuroscience, University of Melbourne, Parkville, VIC 3010, Australia; 2 Florey Institute of Neuroscience and Mental Health, Parkville, VIC 3010, Australia

**Keywords:** amygdala, cFos, engram, fear learning, plasticity, synapse

## Abstract

The lateral amygdala (LA) plays a critical role in the formation of fear-conditioned associative memories. Previous studies have used c-*fos* regulated expression to identify a spatially restricted population of neurons within the LA that is specifically activated by fear learning. These neurons are likely to be a part of a memory engram, but, to date, functional evidence for this has been lacking. We show that neurons within a spatially restricted region of the LA had an increase in both the frequency and amplitude of spontaneous postsynaptic currents (sPSC) when compared to neurons recorded from home cage control mice. We then more specifically addressed if this increased synaptic activity was limited to learning-activated neurons. Using a *fos*-*tau-LacZ* (*FTL*) transgenic mouse line, we developed a fluorescence-based method of identifying and recording from neurons activated by fear learning (*FTL^+^*) in acute brain slices. An increase in frequency and amplitude of sPSCs was observed in *FTL^+^* neurons when compared to nonactivated *FTL^−^* neurons in fear-conditioned mice. No learning-induced changes were observed in the action potential (AP) input-output relationships. These findings support the idea that a discrete LA neuron population forms part of a memory engram through changes in synaptic connectivity.

## Significance Statement

The lateral amygdala (LA) is critical for the formation of associative fear memories, such as those formed by fear conditioning. A subset of neurons in LA are activated by fear conditioning suggesting that they may be part of a memory engram. However, there is no functional evidence to support this view. Electrophysiological characterization of LA neurons shows that the frequency and amplitude of synaptic events increase following fear conditioning. A method based on β-galactosidase as a reporter was developed to locate activated neurons in acute slice. An increase in frequency and amplitude of synaptic events was observed in activated compared to nonactivated neurons supporting the idea that a discrete LA neuron population forms part of a memory engram.

## Introduction

Memory formation has long been hypothesized to occur via enhanced synaptic connectivity between populations of neurons in the brain (Hebb, 1949). While there is much indirect evidence supporting this hypothesis (Thompson, 2005; Josselyn et al., 2015; Sweatt, 2016), direct evidence is lacking because of the difficulty in identifying neurons that are specifically involved in the formation of a particular memory. One model of learning and memory which has facilitated the identification of neurons involved in memory is classical fear conditioning. Fear conditioning is a well-validated model of associative learning, with the lateral amygdala (LA) strongly implicated in the formation of the associative memories (Maren, 2001; Pape and Stork, 2003; Fanselow and Poulos, 2005; Kim and Jung, 2006; LeDoux, 2007; Johansen et al., 2011). Extracellular single-unit recordings of LA neurons during auditory fear conditioning show that a subpopulation of LA neurons alters their rate of tone-evoked firing (Repa et al., 2001). In addition, selective ablation of a subset of CREB-overexpressing neurons in the LA results in the erasure of a conditioned fear memory (Han et al., 2009; Rogerson et al., 2016). These studies suggest that plasticity in only a subset of LA neurons forms part of the memory trace in fear learning.

Immediate early gene expression provides one method of identifying neurons that have been functionally activated during the learning process (Dragunow, 1996). The immediate early gene *c-fos* has been extensively used as a marker of neuronal activation (Knapska et al., 2007) and previous studies have employed *c-fos* regulated expression in the transgenic *fos*-*tau-LacZ* (*FTL*) mouse line to identify functionally activated circuitry in the brain (Murphy et al., 2007; Wilson and Murphy, 2009). Using *FTL* mice, a number of discrete, anatomically defined populations of neurons in different parts of the brain were identified as being specifically activated by fear learning (Wilson and Murphy 2009; Trogrlic et al., 2011; Butler et al., 2015). In particular, a population of learning-activated *FTL^+^* neurons was found within a spatially restricted region of the ventrolateral nucleus of the LA (LAvl). Other studies are also consistent with neurons specifically in LAvl playing a key role in fear memory (Schafe et al., 2000; Bergstrom et al., 2013; Besnard et al., 2014). Thus, where it is established that activation of the ERK1/2 kinase in LA is required for fear memory formation, ERK1/2 activation following fear conditioning occurs predominantly in neurons in LAvl (Schafe et al., 2000), in a similar pattern to that seen with *FTL^+^* neurons.

Given the importance of the LA in the formation of fear memory, the learning-activated *FTL^+^* neurons are prime candidates for direct involvement in fear memory. However, it has not been established if these neurons undergo specific changes in their synaptic or intrinsic properties following activation in fear learning. Such studies may provide further evidence both for involvement of these neurons in fear memory and for the nature of the “memory engram.” In this study, we first trained wild-type mice to simultaneously record the synaptic and firing properties of LAvl neurons after fear learning. Recordings from neurons in brain slices revealed significant increases in the amplitude and frequency of spontaneous synaptic events in a subpopulation of LAvl neurons from fear-conditioned mice compared with neurons from home cage mice. Subsequently, we used the activity-dependent expression of the *FTL* transgene to target recordings to the learning activated neurons in brain slices from trained *FTL* mice. Similar increases in amplitude and number of spontaneous synaptic events recorded from *FTL^+^* neurons were observed, relative to their nonactivated neighbors. We suggest that these synaptic changes, which occur within a specific population of LAvl neurons following learning, are central to the formation of the fear memory engram.

## Materials and Methods

### Animals

All animal procedures were performed in accordance with the University of Melbourne Animal Care Committee’s regulations. Male *FTL^−^* and *FTL^+^* mice, on a C57BL6/J background, aged four to seven weeks, were housed in standard 15 × 30 × 12 cm cages on a 12/12 h light/dark cycle and had food and water supplied *ad libitum*. Two days before the commencement of the experiment, mice were singly housed in cages and relocated to a dedicated behavioral laboratory. In this facility, mice were housed under quiet (60 dB), low-light (15–20 Lux) conditions and a 12/12 h light/dark cycle.

### Context fear conditioning

Fear conditioning was conducted using a computerized system (Clever Sys Inc.). The conditioning chamber (32 × 26 × 21 cm) was contained within a sound-attenuating compartment. The floor of the chamber consisted of stainless steel rods connected to a shocker-scrambler unit capable of delivering electric shocks of defined duration and intensity. The chamber was cleaned with 70% ethanol between training and testing sessions. For fear conditioning, mice were placed in the chamber for 3 min, after which they received a 1.0-mA, 2-s footshock. The mice then remained in the chamber for a further 30 s before being returned to their home cages. For the histochemistry experiment, in addition to the previous training conditions, an immediate shock group was exposed to a 1.0-mA, 2-s footshock immediately on entry into the training chamber. Mice in this group then remained in the training chamber for 3 min and 30 s. The immediately shocked mice did not exhibit any context-associated fear learning when tested. Furthermore, non-learning control mice are not reported to exhibit appreciable *FTL* expression in the LAvl (Trogrlic et al., 2011).

Mice remained in their home cages for 3 h after training, to induce *FTL* expression in activated neurons. Mice were then tested for context-associated fear by placing the mice back in the same training chamber for 3 min. Freezing and moving behavior was recorded, using automated software (Clever Sys Inc.). Mice were anaesthetized immediately after testing. Home cage mice were anaesthetized immediately on removal from their home cages. In total, 13 *FTL^−^* (eight home cage, six context fear-conditioned) and seven *FTL^+^* (all context fear-conditioned) were used.

### Histochemistry

*FTL^+^* neurons can be identified in fixed tissue using β-galactosidase histochemistry. To show learning-induced *FTL^+^* neurons in the LAvl region, context fear-conditioned and immediate shock mice were sacrificed immediately following testing (3 h after training). Brains were removed from the mice and 300-μm coronal slices were cut on a vibratome (VT1200; Leica). Slices were immersion fixed in 4% paraformaldehyde for 10 min and transferred to separate wells containing 400-µl X-gal solution (10 mM potassium ferricyanide, 10 mM potassium ferrocyanide, 40 mM MgCl_2_, and 2 mg/ml 5-bromo-4-chloro-3-indolyl-β-D-galactopyranoside; Roche, Life Sciences) in 0.1 M PBS for 24 h at room temperature, with agitation. The slices were then rinsed with PBS and mounted onto Superfrost+ glass slides (Thermo Fisher Scientific). Sections were left to dry and coverslipped.

### Electrophysiology

Mice were anaesthetized with 2% isofluorane, before decapitation. The brains were quickly removed and placed into ice cold cutting solution (125 mM choline chloride, 2.5 mM KCl, 0.4 mM CaCl_2_, 6 mM MgCl_2_, 1.25 mM NaH_2_PO_4_, 26 mM NaHCO_3_, and 20 mM D-glucose) saturated with carbogen gas (95% O_2_–5% CO_2_), and 300-μm coronal slices were cut on a Vibratome (VT1200; Leica). Slices containing the LA (approximately two slices per mouse) were incubated at room temperature for a minimum of 1 h in artificial CSF (aCSF; 125 mM NaCl, 2.5 mM KCl, 2 mM CaCl_2_, 2 mM MgCl_2_, 1.25 mM NaH_2_PO_4_, 26 mM NaHCO_3_, and 10 mM D-glucose) saturated with carbogen. For *FTL* experiments, 25 µM C_12_-fluorescein-di-(β-D-galactopyranoside) (C_12_FDG, Imagene Green, Thermo Fisher Scientific) was added to the aCSF solution.

For patch clamping experiments, slices were transferred to a submerged chamber and perfused (8 ml/min) with aCSF at room temperature. The LAvl was located, and neurons were visualized with IR-DIC microscopy. Glutamatergic projection neurons were targeted for recording, distinguished from interneurons by large pyramidal-like somata (Sosulina et al., 2006). Electrodes were pulled using a Sutter P-2000 puller (Sutter Instruments) from borosilicate micropipettes (World Precision Instruments) with an initial resistance of around 3–6 MΩ. Electrodes were filled with: 125 mM K-gluconate, 5 mM KCl, 2 mM MgCl_2_, 10 mM HEPES, 4 mM ATP-Mg, 0.3 mM GTP-Na_2_, 10 mM tris-phosphocreatine, and 10 mM EGTA (pH 7.2, 290 OsM). Biocytin (2 mg/ml) was included to allow morphologic examination and verification of *FTL^+^* neurons *post hoc*. Whole-cell patch clamp recordings were made using a MultiClamp 700A amplifier and pClamp acquisition software (Molecular Devices) from neurons visually identified using infrared DIC imaging (BX51, Olympus). For *FTL* experiments, neurons were identified as either *FTL^+^* or *FTL*^–^ by the presence or absence of C_12_FDG fluorescence using a GFP filter set (excitation 457–487 nm and emission 502–538 nm). The number of neurons recorded for the experiment involving the application of C_12_FDG was 13 from *FTL* mice (six *FTL* ^−^, seven *FTL^+^*; all context fear-conditioned). In a subset of these experiments we were able to record at –50 mV (four *FTL*^−^ neurons, six *FTL*^+^ neurons). For the nontargeted experiment, 61 neurons were recorded from wild-type mice (28 from home cage mice, 33 from context fear-conditioned mice). No more than two neurons were recorded from each brain slice.

### Firing properties

Firing properties were recorded in current clamp mode. Bridge balance and capacitance compensation was applied to all recordings. Voltage recordings were filtered at 30 kHz and sampled at 100 kHz. Resting membrane potential was measured and neurons recorded as being more than –55 mV were excluded. A holding current was injected into neurons, if required, setting their holding potential to approximately –65 mV. A current injection/action potential (AP) frequency relationship was established by injecting 30 current steps of 500-ms duration (10-pA incremental steps from –30 to 260 pA). All electrophysiological data were analyzed using AxoGraph X software. To calculate membrane time constant, an exponential function was fit to the voltage trace following a –20-pA current injection for each cell. An automated detection algorithm was used to detect AP with visual confirmation. AP threshold voltage was defined as the voltage at which gradient reached 10 mV/ms^−1^. For the average AP wave form analysis, APs were aligned to the threshold. AP properties measured were peak height, width at 50% of height, rise time (10–90% height), and decay time (100–50%). Peak AP amplitude was measured from threshold to peak. Rheobase was defined as the current injection that first fired an AP.

### Synaptic analysis

Spontaneous postsynaptic currents (sPSCs) were measured in voltage-clamp mode in the absence of any neurotransmitter blockers. Currents were recorded at –70 and –50 mV and sampled at 10 kHz with filtering set at 3 kHz. sPSCs were identified using event detection in Axograph X (Axograph Scientific Software). The detection threshold of sPSCs was set at four times the SD of the noise. Each automatically identified event was manually confirmed. Amplitude and interevent interval (IEI) were calculated and cumulative probability curves constructed using 60-s gap-free recording with at least 25 events per neuron. No series resistance compensation was applied. NBQX (20 µM) and APV (50 µM) were added to ACSF to determine the reversal potential of GABA_A_ receptor-mediated Cl^−^ current (measured from seven wild-type neurons). Synaptic events were recorded at various voltages and their amplitudes measured using the automated method described above. We have not accounted for liquid junction potential in any recordings.

### Statistics and Gaussian peak fitting

All statistical tests were performed with GraphPad software (Prism). Freezing and moving data were analyzed using two-tailed Student’s *t* tests. sPSC IEIs and amplitudes were analyzed using Student’s *t* tests with Welch’s correction, while cumulative probability curves were compared using the Kolmogorov–Smirnov test. IO curves were analyzed using two-way ANOVAs, examining current injection and *FTL* status as sources of variation. AP parameters were compared using unpaired Student’s *t* tests with Welch’s correction. A peak fitting algorithm was applied to the data using an unconstrained nonlinear optimization algorithm to decompose the percentage of recorded neurons versus sPSC frequency and amplitude data into Gaussian peaks (only the number of peaks was specified). The peak fits used the MATLAB script of “findpeak.m” (https://terpconnect.umd.edu/~toh/spectrum/InteractivePeakFitter.htm). Bar graphs presented in the figures represent mean ± SEM.

### Fluorescence immunohistochemistry

Fluorescence immunohistochemistry was used to verify the *FTL* status of recorded and biocytin-filled neurons from *FTL* mice. After patch-clamp experiments, slices were immersion fixed with 4% paraformaldehyde for 1 h. Slices were incubated with blocking solution (3% bovine serum albumin, 0.3% Triton X-100, and 50 µM glycine, diluted in PBS) for 1 h on an orbital shaker, followed by primary antibody solution [chicken anti-β-galactosidase (Abcam, RRID: AB_307210), diluted 1:1000 in 0.1% Triton X-100/PBS] overnight. Slices were then exposed to secondary antibody solution (goat anti-chicken Alexa Fluor 488 1:500, Streptavidin 594 1:500, diluted in 0.1% Triton X-100/PBS) for 6 h. Slices were mounted in PBS onto Superfrost+ glass slides (Thermo Fisher Scientific), and coverslipped using fluorescent mounting medium (Dako). Slides were visualized using a confocal microscope (LSM5, Carl Zeiss) equipped with a 63× oil-immersion objective (NA 1.4). *FTL* expression was detected after illumination via an Argon laser at 488 nm (emission bandpass filter 515–530 nm). Biocytin fluorescence was excited via a DPSS laser at 561 nm and the emission collected using a bandpass filter (575–615 nm). Dual channel z-stack images were taken (1024 pixels × 1024 pixels × 55 planes, voxel size 0.12 × 0.12 × 0.43 µm) and then projected using maximum intensity (ImageJ, NIH).

## Results

### LAvl neurons display increased spontaneous postsynaptic potential frequency and amplitude following fear conditioning

**Figure 1.**
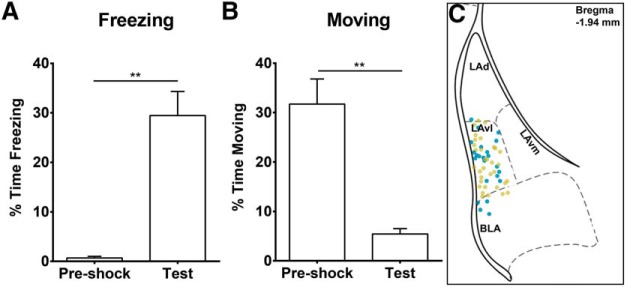
Recorded neurons in the LAvl region from fear-conditioned and home cage mice. fear-conditioned mice were shown to have acquired a context-fear memory by a significant increase in freezing behavior (*p* = 0.0018; ***A***) and a significant decrease in moving behavior (*p* = 0.0015, *n* = 6; ***B***). ***C***, Cartoon illustrating the position of whole-cell recorded neurons within the LAvl region of the amygdala from fear-conditioned mice and home cage control mice. Blue markers = home cage; yellow markers = fear-conditioned trained mice; ** indicates statistical significant change.

To see whether fear learning had any effects on the electrophysiological characteristics of neurons in LA, a group of wild-type mice were fear-conditioned for comparison with a home cage control group. Consistent with having learnt contextual fear, the fear-conditioned mice had significantly increased freezing episodes and reduced movement (Fig. 1*A*,*B*; pre-shock freezing *M* = 0.66, *SD* = 0.82; test freezing *M* = 29.5, *SD* = 12; *t*_(5)_ = 6, *p* = 0.0018; pre-shock moving *M* = 32, *SD* = 13; test moving *M* = 5.4, *SD* = 2.7; *t*_(5)_ = 6.3, *p* = 0.0015). A small subset of neurons within the LAvl has been previously identified to be specifically activated by fear conditioning (Wilson and Murphy, 2009; Trogrlic et al., 2011; Butler et al., 2015). Whole-cell recordings were made from neurons in this LAvl subnucleus region from fear-conditioned and home cage control mice as marked in Figure 1*C*. There were no differences in the recorded passive neuron properties as reported in Table 1.

**Table 1.** Passive neuron properties

	Untargeted neurons				Targeted neurons			
	HC	FC	*t*	*p*	*FTL*^+^	*FTL*^−^	*t*	*p*
*N*	28	33	-	-	7	6	-	-
Input resistance (MΩ)	273 ± 139	281 ± 143	1.1	0.25	177 ± 59	162 ± 86	0.73	0.73
Membrane potential (mV)	–66 ± 7	–66 ± 6	0.06	0.96	–66 ± 4	–65 ± 8	0.56	0.59
Membrane time constant (ms)	51 ± 16	56 ± 16	0.5	0.60	47 ± 10	42 ± 10	1.21	0.25

**Figure 2.**
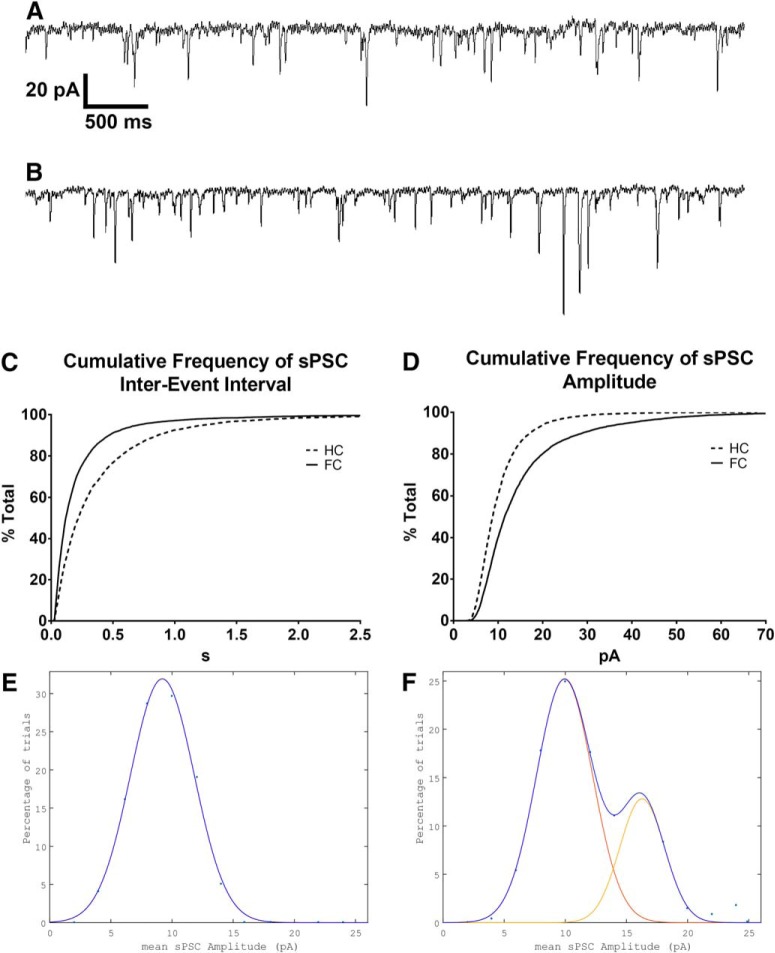
Learning induces increased synaptic activity in a subpopulation of LAvl neurons. Raw traces of sPSCs from (***A***) home cage mice (HC) and (***B***) fear-conditioned mice (FC). ***C***, The cumulative frequency graph of the IEI of sPSCs shows a significant left shift in fear-conditioned mice compared to home cage controls (Kolmogorov–Smirnov test *p* < 0.0001). ***D***, The cumulative frequency graph of the amplitude of sPSCs shows a significant right shift in fear-conditioned mice, compared to home cage controls (Kolmogorov–Smirnov test *p* < 0.0001, *n* = 28 HC and *n* = 33 FC). ***E***, A Gaussian fit to the frequency histogram of the sPSC amplitude of neurons from home cage controls shows a single peak. ***F***, A second peak is evident in fear-conditioned mice.

We measured sPSCs at a –70-mV holding potential to evaluate excitatory/inhibitory synaptic network activity onto these neurons (Fig. 2*A*,*B*). The cumulative frequency plot of the IEI for sPSCs showed a significant left-shift of the curve for neurons recorded from fear-conditioned mice compared to home cage mice (Fig. 2*C*; Kolmogorov–Smirnov D = 0.222, *p* < 0.0001, averaged data: HC *M* = 0.38 s, *SD* = 0.5 s; FC *M* = 0.23 s, *SD* = 0.38 s; *t*_(6239)_ = 17.1, *p* < 0.0001). Furthermore, the cumulative frequency plot of the sPSC amplitude showed a significant right shift for neurons recorded from fear-conditioned mice compared to home caged mice (Fig. 2*D*; Kolmogorov–Smirnov D = 0.2159, *p* < 0.0001, averaged data: HC *M* = 10.4 pA, *SD* = 5.9 pA; FC *M* = 15.3 pA, *SD* = 11.7 pA; *t*_(12210)_ = 30.8, *p* < 0.0001). These findings are consistent with a change in synaptic properties of neurons within the LAvl after fear conditioning. However, based on previous reports identifying activated neurons histochemically, only a subset of neurons within the LAvl are expected to have altered properties (Wilson and Murphy, 2009; Trogrlic et al., 2011; Butler et al., 2015). To explore this further, we applied a Gaussian peak-fitting algorithm to the average sPSC amplitude of each cell recorded from either fear-conditioned or home-caged mice (Fig. 2*E*,*F*). The algorithm identified a single Gaussian distribution peaking at 9.2 pA for home-caged mice but identified two Gaussian peaks (at 9.9 and 16 pA) in conditioned mice. The Gaussian peak-fitting algorithm was also applied to sPSC frequency data. The algorithm identified three peaks (at 2.5, 5, and 10 Hz), which were at similar frequencies in each condition (data not shown). However, the proportions of cells that fell within each peak differed between conditions. The Gaussian fit to the highest frequency accounted for 25% of cells in the fear learning mice but only 4% in home-caged mice. These empirical observations are consistent with the idea that only a subpopulation of neurons in LAvl exhibit altered properties following fear learning (Repa et al., 2001; Sehgal et al., 2014), and with previous findings that fear learning activates a specific subpopulation of neurons in LAvl (Wilson and Murphy, 2009; Trogrlic et al., 2011; Butler et al., 2015). We next used an approach that allowed the electrophysiological recording of neurons specifically activated by fear conditioning.

### Identification of *FTL^+^* learning-specific neurons in LAvl

**Figure 3.**
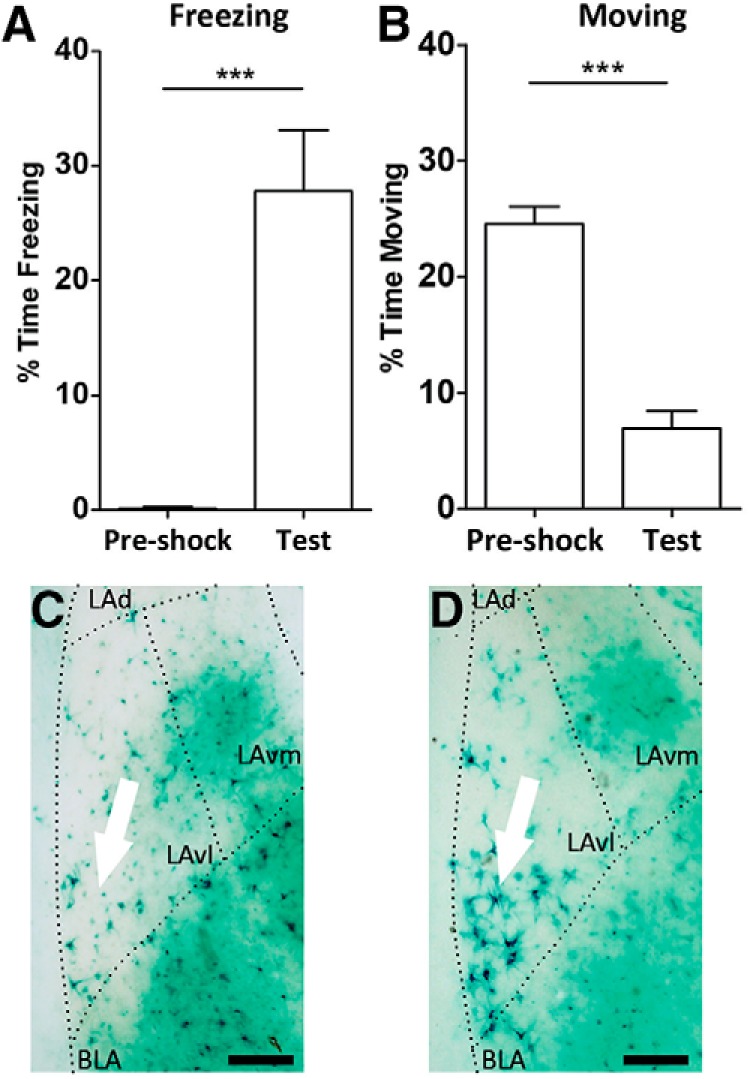
Restricted expression of *FTL*^+^ neurons in thick brain slices following fear conditioning. Context fear-conditioned mice acquired a fear memory illustrated by increased (***A***) freezing relative to pre-shock levels (*p* = 0.0002) and (***B***) decreased movement (*p* < 0.0001, *n* = 7). ***C***, ***D***, Micrograph of the amygdala region of an *FTL* mouse stained with x-gal histochemistry following immediate shock training (non-learning control; ***C***) and following delayed shock training (fear-conditioned; ***D***), showing an increase in *FTL*^+^ neurons specifically in the LAvl. Scale bars in ***C***, ***D***: 150 µm. *** indicates statistically significant change.

To test the electrophysiological properties of activated neurons, we developed a method to label and identify them in live brain slices based on the *FTL* mouse. The *FTL* mouse consists of a *tau-LacZ* transgene driven by the *c-fos* promoter, which results in expression of β-galactosidase throughout neurons in which *c-fos* has been expressed. First, we determined if the expected pattern of learning activated neurons in LAvl could be identified in the thick brain slices used for electrophysiology. For this, *FTL* mice were context fear-conditioned and then tested for context-associated fear 3 h later. Mice trained in fear conditioning spent a significantly higher percentage of time freezing during this testing session than during the pre-shock period in training (Fig. 3*A*; pre-shock *M* = 28, *SD* = 14*;* Test *M* = 0.14, *SD* = 0.38; *t*_(12)_ = 5.2, *p* = 0.0002). The trained mice also spent a significantly lower percentage of time moving during testing when compared to the pre-shock training session (Fig. 3*B*; pre-shock *M* = 6.9, *SD* = 4; Test *M* = 25, *SD* = 4; *t*_(12)_ = 8.2, *p* < 0.0001;). Conditioned mice had therefore acquired a context-associated fear memory. Thick brain slices prepared for electrophysiological analysis were taken from these mice and stained for β-galactosidase. These slices showed a clear pattern of *FTL* activation in a small subset of neurons along the border of the LAvl, with only a few *FTL^+^*neurons visualized in equivalent sections from immediate shock mice (Fig. 3*C*,*D*). The pattern of *FTL^+^* neurons we observed in conditioned mice is consistent with the pattern of *FTL* activity described in previous studies (Wilson and Murphy, 2009; Trogrlic et al., 2011; Butler et al., 2015).

**Figure 4.**
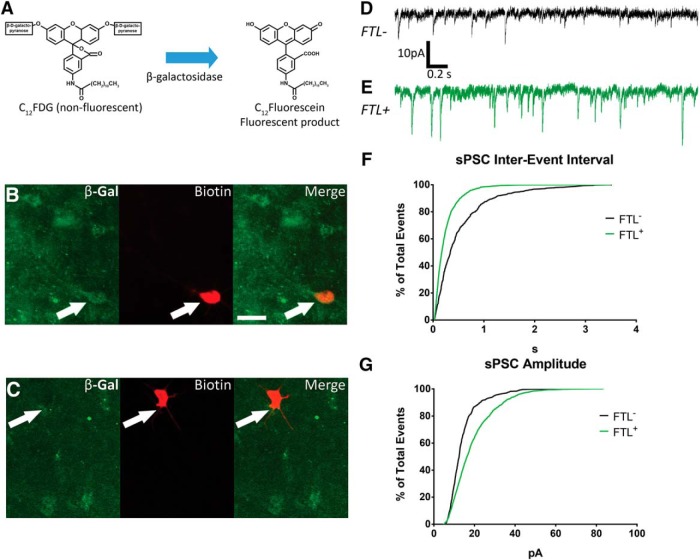
FTL^+^ neurons in the LAvl show increases in sPSC frequency and amplitude. ***A***, C_12_FDG is converted to the fluorescent product C_12_-fluorescein by β-galactosidase, enabling the identification of *FTL^+^* neurons in acute brain slices. *FTL^+^* (***B***) and *FTL*^−^ (***C***) neurons identified *post hoc* by double labeling for β-galactosidase and biotin from intracellular recording. Representative voltage-clamp traces from (***D***) *FTL*^−^ and (***E***) *FTL^+^*****neurons in the LAvl of context fear-conditioned mice. ***F***, Cumulative frequency histogram of the IEIs of recorded sPSCs from *FTL^+^* and *FTL*^−^ neurons (Kolmogorov–Smirnov test *p* < 0.0001). ***G***, Cumulative frequency histogram of the amplitudes of recorded sPSCs from *FTL^+^* and *FTL*^−^ neurons (Kolmogorov–Smirnov test *p* < 0.0001, *n* = 6 *FTL^−^* and *n* = 7 *FTL^+^*). Scale bars in ***B***, ***C***: 25 µm.

We then developed a novel methodology using the fluorogenic β-galactosidase substrate C_12_FDG as a marker of *FTL* expressing neurons in acute brain slices (Fig. 4*A*). C_12_FDG is a β-galactosidase substrate that is cell permeable (Zhang et al., 1991; Plovins et al., 1994), and in the presence of β-galactosidase, the galactopyranose moieties are cleaved to yield a fluorescent product (Rotman et al., 1963). *FTL^+^* neurons in the LAvl of context- fear-conditioned mice were identified based on their fluorescent signal. *Post hoc* analysis of biocytin-filled neurons confirmed their *FTL* identity: the biocytin signal overlapped with that of β-galactosidase for *FTL^+^* neurons while, if the neuron was *FTL*^−^, only the biocytin signal was seen (Fig. 4*B*,*C*). These findings demonstrated that it was possible to identify *FTL^+^*neurons in acute brain slices using C_12_FDG, which could then be targeted for whole-cell patch clamp recordings. We observed no differences in the recorded passive properties of *FTL*^+^ and *FTL*^−^ neurons as reported in Table 1.

### Increased spontaneous synaptic activity in learning-specific neurons

To examine synaptic changes that may have occurred following context fear conditioning, we recorded sPSCs from the *FTL^+^*and *FTL*^−^ neurons in the LAvl of the fear-conditioned mice at a holding potential of –70 mV (Fig. 4*D*,*E*). The cumulative frequency plot of the IEI for sPSCs showed a significant left-shift of the curve for *FTL^+^*neurons compared to *FTL*^−^ neurons (Fig. 4*F*; Kolmogorov–Smirnov D = 0.29, *p* < 0.0001, averaged data: *FTL*^−^ *M* = 0.52 s, *SD* =0.57; *FTL*^+^ *M* = 0.25 s, *SD* = 0.25; *t*_(562)_ = 10.7, *p* < 0.0001). Furthermore, the cumulative frequency plot of the sPSC amplitude showed a significant right shift of the curve for *FTL^+^* neurons compared to *FTL*^−^ neurons (Fig. 4*G*; Kolmogorov–Smirnov D = 0.27, *p* < 0.0001, *FTL*^−^ *M* = 14 pA, *SD* = 8; *FTL*^+^ *M* = 19 pA, *SD* = 11; *t*_(1228)_ = 11.0, *p* < 0.0001). Together, these observations illustrate an increase in both the frequency and amplitude of synaptic activity onto *FTL^+^* neurons when compared with neighboring *FTL*^−^ neurons.

**Figure 5.**
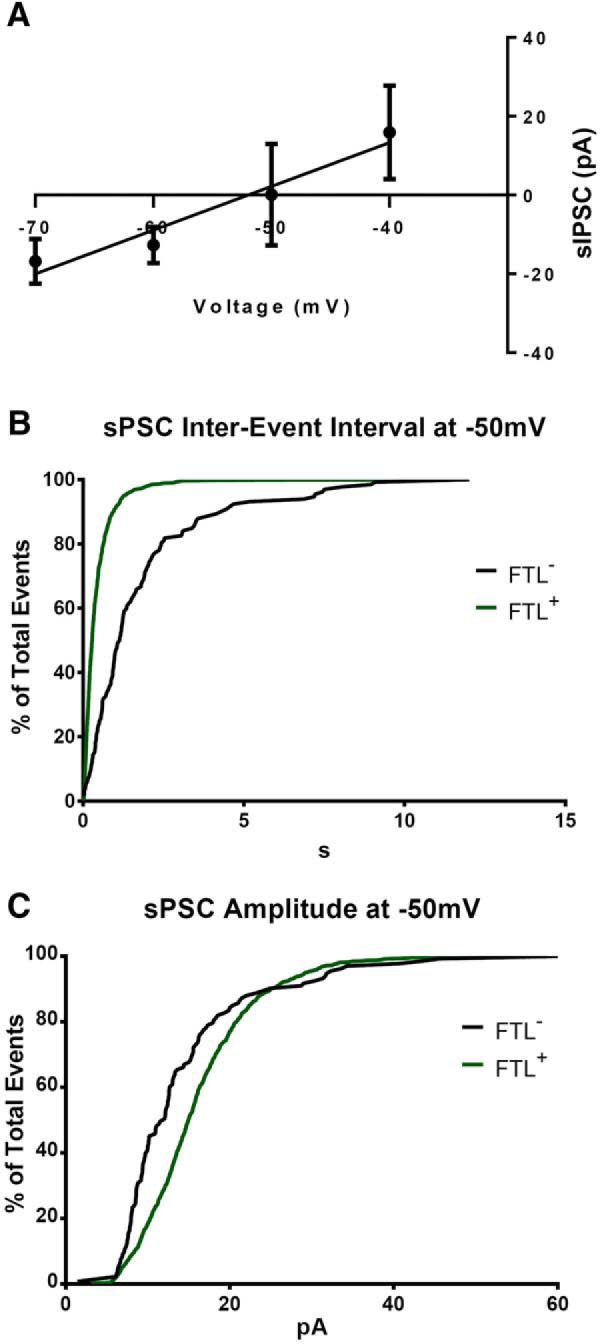
Increases in the spontaneous excitatory synaptic current event amplitude and frequency. ***A***, Average spontaneous inhibitory PSC (sIPSC) amplitude at varying holding potentials in the presence of NBQX and APV from neurons in the mouse LA, showing a reversal potential of Cl^−^ of approximately –50 mV (*n* = 7). ***B***, The cumulative frequency graph of the IEI of sPSCs from *FTL^+^* and *FTL*^−^ neurons at –50 mV (Kolmogorov–Smirnov test *p* < 0.0001). ***D***, The cumulative frequency graph of the amplitude of sPSCs at –50 mV *FTL^+^*and *FTL*^−^ neurons (Kolmogorov–Smirnov test *p* < 0.0001, *n* = 4 *FTL^−^* and *n* = 6 *FTL^+^*).

In a subset of these neurons we were able to record sPSC at a holding potential of –50 mV, which is near the reversal potential of Cl^−^ (Fig. 5*A*). The pattern of reduced sPSCs IEI (Fig. 5*B*; Kolmogorov–Smirnov D = 0.53, *p* < 0.0001; averaged data: *FTL*^−^ *M* = 1.8 s, *SD* = 2; *FTL*^+^ *M* = 0.43 s, *SD* = 0.50; *t*_(133)_ = 7.6, *p* < 0.0001) and increased amplitude (Fig. 5*C*; Kolmogorov–Smirnov D = 0.273, *p* < 0.0001; averaged data: *FTL*^−^ *M* = 14 pA, *SD* = 8; *FTL*^+^ *M* = 16 pA, *SD* = 7; *t*_(164)_ = 3.0, *p* = 0.0027) was still observed under these conditions, suggesting that the observed increase in synaptic activity was, at least in part, through excitatory transmission. Together, these findings indicate that the neurons in LAvl activated to express *FTL* by fear conditioning have undergone significant learning-induced synaptic plasticity.

### Firing properties of LAvl neurons after fear-induced learning

**Figure 6.**
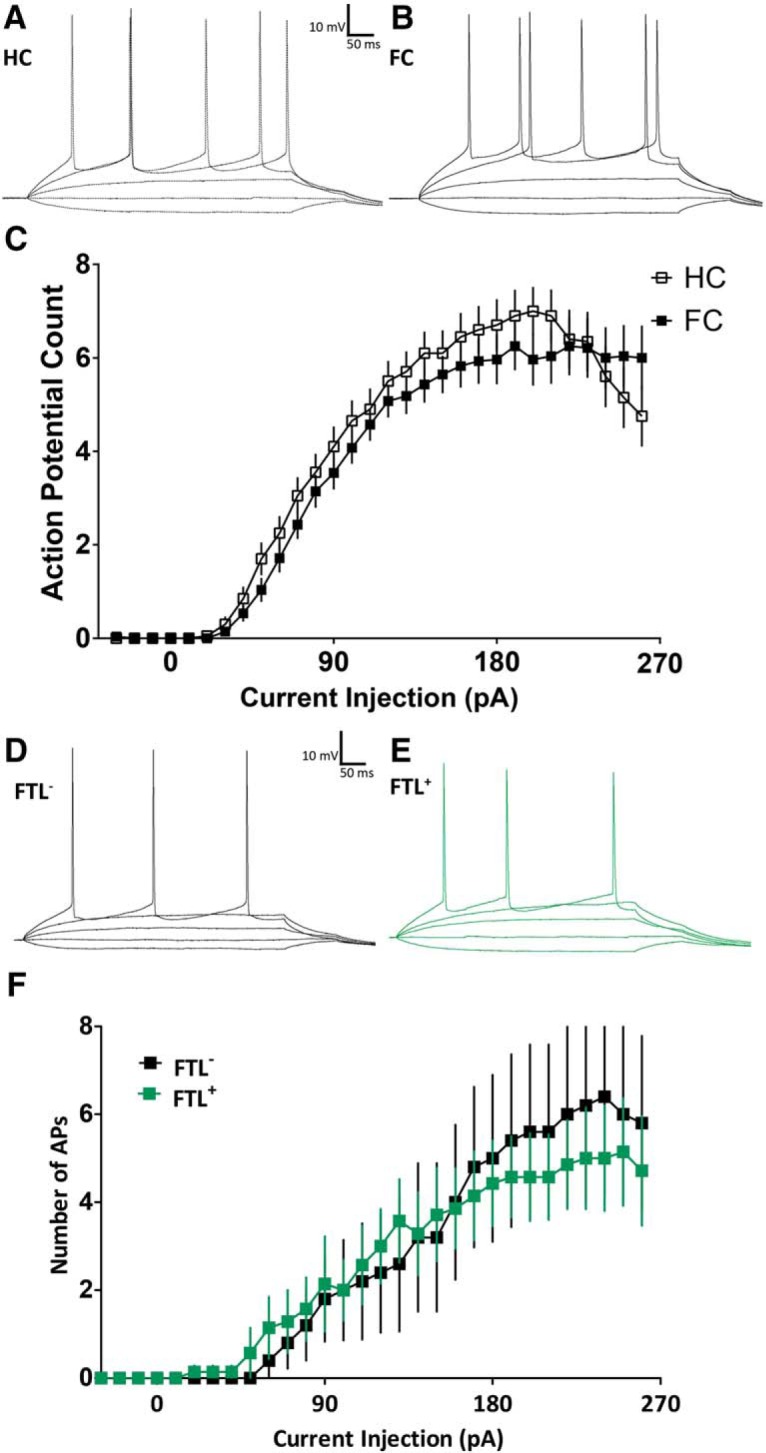
LAvl neurons show no differences in firing properties following fear conditioning. ***A***, ***B***, Representative traces from current-clamped (***A***) home cage (HC) and (***B***) fear-conditioned (FC) neurons in the LAvl. ***C***, Mean AP count versus current injection for neurons from context fear-conditioned mice and from untrained home cage mice (*n* = 28 HC and *n* = 33 FC). Representative traces from current-clamped (***D***) *FTL*^−^ and (***E***) *FTL^+^* neurons in the LAvl of context fear-conditioned mice. ***F***, Plot of the mean AP count versus current injection for *FTL^+^* and *FTL*^−^ neurons from fear-conditioned mice (*n* = 6 *FTL^−^* and *n* = 7 *FTL^+^*).

Analysis of the firing properties of LAvl neurons in wild type experimental mice reveals a similar input/output relationship (Fig. 6*A–C*) and rheobase (home caged (HC) *M* = 60 pA, *SD* = 40 pA; fear conditioned (FC) *M* = 57 pA, *SD* = 18 pA; *t*_(29)_ = 0.34, ns) between conditioned and home cage control mice. Fear conditioning did induce significant changes in the shape of LAvl APs, as measured by the peak height, peak width, rise parameter, and decay parameter (Table 2).

**Table 2.** AP properties of LAvl neurons

	Untargeted neurons				Targeted neurons			
	HC	FC	*t*	*p*	*FTL* ^+^	*FTL* ^−^	*t*	*p*
*N*	28	33	-	-	7	6	-	-
AP height (mv)	88 ± 7	83 ± 9	2.0	0.047	89 ± 5	85 ± 14	0.62	0.55
AP width (at 50% height, ms)	2.1 ± 0.3	2.3 ± 0.3	2.8	0.007	2.0 ± 0.3	2.1 ± 0.5	0.50	0.63
Rise time (10–90% peak height, ms)	0.6 ± 0.1	0.7 ± 0.1	3.6	0.0007	0.6 ± 0.1	0.6 ± 0.2	0.039	0.97
Decay time (100–50% peak height, ms)	1.5 ± 0.2	1.7 ± 0.2	2.3	0.028	1.5 ± 0.3	1.6 ± 0.4	0.33	0.75

Firing properties of *FTL*^−^ and *FTL^+^* neurons recorded from fear-conditioned mice were similar with no differences in the input/output relationship (Fig. 6*D–F*) or rheobase (*FTL^+^ M* = 87 pA, *SD* = 51 pA; *FTL*^−^ *M* = 104 pA, *SD* = 44 pA; *t*_(10)_ = 0.59, ns). There was also no difference in the shape of APs produced by *FTL^+^* and *FTL*^−^ neurons, as measured by the peak height, peak width, rise parameter, and decay parameter (Table 2). It should be noted, given the low number of recorded neurons, that small changes in AP properties may not have been detected.

## Discussion

Previous studies identified a spatially discrete population of neurons within LAvl which was specifically activated by fear conditioning (Wilson and Murphy, 2009; Trogrlic et al., 2011; Butler et al., 2015). These neurons are, therefore, specifically implicated in fear memory formation. To look for memory related electrophysiological changes within LAvl, we first recorded from neurons in wild-type mice following fear conditioning. These recordings revealed a subpopulation of neurons in LAvl that exhibited increases in sPSC frequency and amplitude compared with neurons from home-caged mice. to target the discrete population of neurons within LAvl which was specifically activated by fear conditioning, we then developed a method of identifying and recording from *FTL^+^*neurons in *FTL* brain slices labeled for β-galactosidase. Neurons that exhibited learning-specific β-galactosidase activation displayed increased sPSC frequency and amplitude compared to neighboring non-labeled neurons. Recordings of a subset of these labeled neurons at the reversal potential of Cl^−^ suggest that, at least in part, the increases in amplitude and frequency of synaptic events are mediated through excitatory transmission. These findings provide evidence that increases in synaptic strength in a small, spatially discrete population of neurons in LA form part of the fear memory engram.

The fluorogenic β-galactosidase substrate C_12_FDG has been previously used primarily in flow cytometry applications, including for the analysis of mammalian cell types (Gu et al., 1993; Miao et al., 1993; Beattie et al., 1994). However, the use of C_12_FDG to identify cells for electrophysiological analysis has not been previously reported. A number of other fluorogenic β-galactosidase substrates exist (Haugland and Johnson, 1993) however C_12_FDG was chosen as it is nontoxic, well retained by cells, and able to passively enter mammalian cells in an aqueous environment (Zhang et al., 1991; Plovins et al., 1994). Our study highlights the use of C_12_FDG as a robust method of identifying labeled neurons expressing any construct that uses β-galactosidase expression as a marker.

Two recent studies investigated the synaptic properties of *Arc* ^+^ neurons in LA after fear conditioning (Nonaka et al., 2014; Gouty-Colomer et al., 2016), based on the premise that *Arc* expression is indicative of a neuron’s involvement in fear learning and memory. Nonaka and colleagues report an increase in the frequency of synaptic currents on *Arc* ^+^ neurons compared to neighboring *Arc*^−^ neurons in conditioned mice (Nonaka et al., 2014). Furthermore, Gouty-Colomer and colleagues also demonstrate synaptic plasticity with increased evoked excitatory currents in *Arc* ^+^ neurons postlearning (Gouty-Colomer et al., 2016). These increases in synaptic event frequency and amplitude were comparable to those observed in the current study. However, it is worth noting that *Arc* expression in these studies may not have been indicative of a neuron’s involvement in memory since there were substantial numbers of *Arc* ^+^ neurons in the non-learning controls, and fear learning stimulated only a small (Gouty-Colomer et al., 2016) or insignificant increase (Nonaka et al., 2014) in *Arc* expression. Thus, the synaptic changes observed in *Arc* ^+^ neurons may have been associated with either learning or non-learning stimuli, such as footshock. In contrast, the LAvl neurons from which we recorded express *FTL* only following fear learning with an average 4-fold increase in *FTL* ^+^ neurons in trained mice compared to unpaired or shock controls (Wilson and Murphy 2009; Trogrlic et al., 2011; Butler et al., 2015). We can thus directly argue that the synaptic changes we observed occurred in neurons that were specifically involved in fear learning.

Our findings of synaptic plasticity can be incorporated into a model of fear learning and memory within the LA as follows. Both context and auditory fear learning induce *c-fos* in a small subset of LAvl neurons (Butler et al., 2015). Given that the LA is crucial for the formation of the association between the conditioned and unconditioned stimuli (Fanselow and LeDoux, 1999; LeDoux, 2000; Blair et al., 2001; Maren, 2001, 2005), neurons involved in fear memory in the LA are possibly a site of convergence of these stimuli. Tracing studies from regions of the thalamus which deliver conditioned stimuli and unconditioned stimuli to the LA show extensive projections to LAvl (LeDoux, 2000). These findings are thus consistent with conditioned stimuli and unconditioned stimuli inputs converging on LAvl neurons, with associative firing of these two pathways resulting in the induction of synaptic plasticity during fear learning. In this model, these LAvl neurons project to downstream regions of the brain which control fear pathways and which are intrinsically activated during signaling from aversive unconditioned stimuli (Fanselow and LeDoux, 1999; LeDoux, 2000; Blair et al., 2001; Maren, 2001, 2005). We propose that the key synaptic changes on these neurons occur at the conditioned stimuli inputs which were activated during fear conditioning and would result in an altered response of these neurons to the conditioned stimuli. This altered response, such as increased frequency and/or amplitude of sPSC, would increase the probability of AP firing and consequently activate the downstream fear pathways in the absence of a unconditional stimulus. After fear learning, the animal would thus display a fear response when exposed to the conditioned stimuli alone. While key aspects of this model need to be examined and tested, it provides a reasonable explanation for how the changes we observed within a small population of neurons in LAvl may underlie part of fear learning and memory.

A number of recent studies have proposed an alternative model of fear memory, whereby neurons in the BLA are incorporated into the memory trace based on their excitability directly before the time of training (Han et al., 2007; Zhou et al., 2009; Gouty-Colomer et al., 2016; Rogerson et al., 2016). Viral-mediated overexpression of CREB in LA neurons has been shown to result in increased neuronal excitability, and transfected neurons were more likely to form part of the fear memory trace (Zhou et al., 2009). Furthermore, optogenetic inactivation of these CREB-overexpressing neurons in the BLA results in attenuation of an acquired fear memory (Rogerson et al., 2016). We find no evidence of changes in the AP input-output relationship of *FTL^+^* and *FTL*^−^ neurons. This finding differs from that of Gouty-Colomer and colleagues, who demonstrated that *Arc* ^+^ neurons had increased AP input-output gain (Gouty-Colomer et al., 2016). The basis of this discrepancy is unclear although it could reflect the relatively small number of neurons recorded. Additionally, EGTA in our internal solution may mask conditioning-associated changes in neuronal firing due to its effects on calcium-activated currents. We have also labeled neurons using a different marker (*c-fos*, cf. *Arc*), and only neurons within the LAvl were targeted, which is a more anatomically restricted region than the wider BLA. Interestingly, we do see subtle changes in AP morphology at a population level in the LAvl of fear-conditioned mice. This was not observed in our targeted approach with no difference in AP morphology observed between *FTL^+^* and *FTL^−^* neurons. The physiologic relevance of this is unclear and warrants future investigation.

In summary, we show that synaptic changes occur specifically on neurons activated by context fear conditioning in the LAvl, with no differences in intrinsic neuronal excitability being detected. These findings suggest a model of fear learning whereby learning-induced synapse modification occurs on neurons within an anatomically restricted region of the LA.

## Synthesis

Reviewing Editor: Quentin Pittman, University of Calgary Decisions are customarily a result of the Reviewing Editor and the peer reviewers coming together and discussing their recommendations until a consensus is reached. When revisions are invited, a fact-based synthesis statement explaining their decision and outlining what is needed to prepare a revision will be listed below. The following reviewer(s) agreed to reveal their identity: Newton Canteras, John Power.

The manuscript examines synaptic strength in lateral amygdala neurons that are “activated” during fear conditioning. The lateral amygdala is a critical brain region for the formation of associative fear memories, including Pavlovian fear conditioning. Previous research has identified several neurophysiological changes in the lateral amygdala following fear conditioning, including an enhancement of the strength of synaptic inputs, increased intrinsic neuronal excitability, and increased expression of immediate early genes such as c-fos. In the current manuscript the authors use a transgenic mouse to fluorescently label neurons expressing the activity dependent transcription factor c-fos, allowing targeted electrophysiological recordings from these neurons. The authors report that following fear conditioning the amplitude and frequency of spontaneous synaptic events is greater in c-fos expressing lateral amygdala neurons than in non-fos expressing neurons. They suggest that these neurons are a critical component of the fear conditioning engram. Overall, the reviewers and I find this to be an interesting study that contributes to our knowledge of memory formation. However, there are concerns about the small number of neurons from FTL mice in the study (12 neurons, 5 FTL- and 7 FTL+) and the conclusions made from the data particularly from negative findings. More detail about some aspects of the methods and result is also needed to enable the data to be interpreted. Major and minor comments are listed below.

1. The methodology underlying sPSC cumulative probability curve construction lacks detail. How many events were used per neuron?

2. Exact p-values and the number of neurons per group should be reported for each comparison. It is unclear how many neurons were used to generate the data in figure 5, one of the most critical figures in the manuscript.

3. Authors should add a column or scatter plots to complement the cumulative histograms.

4. There is concern that soma size is not sufficient to distinguish between projection and interneurons in the lateral amygdala. The authors have collected the information on the action potential and should also use AP width to distinguish between cell types.

5. The authors should report input resistance, resting membrane potential, and membrane time constant values.

6. Given the limited sample size the authors should use caution when discussing the lack of firing property differences between FTL+ and FTL- neurons. Additionally, the 10 mM EGTA in the internal solution may mask conditioning-associated changes in neuronal firing due to EGTA's effect on calcium-activated currents.THis should be acknowledged.

7. Reported firing properties need units

Minor

1. Line 57. “Other studies are also..” - The referenced studies should be cited.

2. Suggest a table of passive membrane properties and firing properties.

## Author Response

The manuscript examines synaptic strength in lateral amygdala neurons that are “activated” during fear conditioning. The lateral amygdala is a critical brain region for the formation of associative fear memories, including Pavlovian fear conditioning. Previous research has identified several neurophysiological changes in the lateral amygdala following fear conditioning, including an enhancement of the strength of synaptic inputs, increased intrinsic neuronal excitability, and increased expression of immediate early genes such as c-fos. In the current manuscript the authors use a transgenic mouse to fluorescently label neurons expressing the activity dependent transcription factor c-fos, allowing targeted electrophysiological recordings from these neurons. The authors report that following fear conditioning the amplitude and frequency of spontaneous synaptic events is greater in c-fos expressing lateral amygdala neurons than in non-fos expressing neurons. They suggest that these neurons are a critical component of the fear conditioning engram. Overall, the reviewers and I find this to be an interesting study that contributes to our knowledge of memory formation. However, there are concerns about the small number of neurons from FTL mice in the study (12 neurons, 5 FTL- and 7 FTL+) and the conclusions made from the data particularly from negative findings. More detail about some aspects of the methods and result is also needed to enable the data to be interpreted. Major and minor comments are listed below. 1. The methodology underlying sPSC cumulative probability curve construction lacks detail. How many events were used per neuron?

Cumulative probability curves were constructed using all events recorded in a standardised 60s period from each neuron, with at least 25 events per neuron represented. These details have been added to the Materials and Methods section at line 183-184.

2. Exact p-values and the number of neurons per group should be reported for each comparison. It is unclear how many neurons were used to generate the data in figure 5, one of the most critical figures in the manuscript.

Exact p-values have been added for each significant comparison, except where the p-value is &lt; 0.0001 (the statistics software package used does not provide exact values below this level). Some incorrect D values for KS analyses have also been corrected. The number of neurons used to generate Figure 5 has been added to the methods and figure legends (line 154, 186 and 569).

3. Authors should add a column or scatter plots to complement the cumulative histograms.

We felt that adding these would have resulted in the figure being too cluttered and less clear and have instead added mean and SD values in the text of the Results (p11, p14). However, we are happy to add this data to the figures if the reviewer thinks it is necessary.

4. There is concern that soma size is not sufficient to distinguish between projection and interneurons in the lateral amygdala. The authors have collected the information on the action potential and should also use AP width to distinguish between cell types.

All neurons included in analysis had comparable peak half-widths (M = 2.18 ms, SD = 0.30 ms). No neurons included in the analysis had a peak half-width of less than 1.6 ms. These data suggest no interneurons were included in the analysis (which typically have much narrower AP peaks). The observed membrane time constants of recorded neurons (40-50 ms, Table 1) also suggest that the analysis included primarily projection neurons, given that interneurons typically show membrane time constants of ~10 ms (Kaneko et al., 2008). Furthermore, previous studies have shown that all FTL+ neurons in the LAvl are glutamatergic (Wilson and Murphy, 2009).

5. The authors should report input resistance, resting membrane potential, and membrane time constant values.

Input resistance, resting membrane potential and membrane time constants have been added as a table (Table 1).

6. Given the limited sample size the authors should use caution when discussing the lack of firing property differences between FTL+ and FTL- neurons. Additionally, the 10 mM EGTA in the internal solution may mask conditioning-associated changes in neuronal firing due to EGTA's effect on calcium-activated currents. This should be acknowledged.

2

We agree with this and have added a sentence about the low numbers in both the Results (334-35) and Discussion sections. We have also acknowledged, in the Discussion, that EGTA may be a confound (ln 417-20). 7. Reported firing properties need units These have been added. Minor 1. Line 57. “Other studies are also..” - The referenced studies should be cited. References cited. 2. Suggest a table of passive membrane properties and firing properties.

We have added a table of both the passive and active properties (Table 1 and Table 2).

References

Kaneko K, Tamamaki N, Owada H, Kakizaki T, Kume N, Totsuka M, Yamamoto T, Yawo H, Yagi T, Obata K, Yanagawa Y (2008) “Noradrenergic excitation of a subpopulation of GABAergic cells in the basolateral amygdala via both activation of nonselective cationic conductance and suppression of resting K+ conductance: a study using glutamate decarboxylase 67-green fluorescent protein knock-in mice.” Neuroscience 157(4):781-97

Wilson YM, Murphy M (2009) “A discrete population of neurons in the lateral amygdala is specifically activated by contextual fear conditioning.” Learn Mem 16(6):357-61
